# Spatial distribution and interactions between mosquitoes (Diptera: Culicidae) and climatic factors in the Amazon, with emphasis on the tribe Mansoniini

**DOI:** 10.1038/s41598-022-20637-2

**Published:** 2022-09-28

**Authors:** Cecilia Ferreira de Mello, Ronaldo Figueiró, Rosemary Aparecida Roque, Daniele Aguiar Maia, Vânia da Costa Ferreira, Anthony Érico Guimarães, Jeronimo Alencar

**Affiliations:** 1 Laboratório de Diptera, Instituto Oswaldo Cruz, FIOCRUZ, Avenida Brasil, n. 4365, Manguinhos, Rio de Janeiro, RJ CEP 21040-360 Brazil; 2 Programa de Pós-Graduação em Biologia Animal, UFRRJ, Rd BR 465, Km 7, Seropédica, RJ CEP 23897-000 Brazil; 3 Departamento de Biologia, Faculdade de Ciências Biológicas e Saúde, Universidade do Estado do Rio de Janeiro, Avenida Manuel Caldeira de Alvarenga, 1203, Campo Grande, Rio de Janeiro, RJ CEP 23070-200 Brazil; 4 Instituto Nacional de Pesquisas da Amazônia, INPA, Avenida André Araújo, n. 2936, Petrópolis, Manaus, Amazonas CEP 69067-375 Brazil; 5 Energia Sustentável do Brasil, ESBR, Rodovia BR- 364, KM 824 S/N, Distrito de Jaci Paraná, Porto Velho, Rondônia 76840-000 Brazil

**Keywords:** Entomology, Ecology, Zoology

## Abstract

This work aimed to evaluate the spatial distribution of mosquitoes in different seasonal periods and the interaction between climatic factors and the abundance of mosquitoes, especially those belonging to the tribe Mansoniini in the area surrounding the Amazon hydroelectric production region (Jirau-HP) of Rondônia state, Brazil. Mosquito specimens were collected in May, July, October, and December 2018, and April, July, September, and November 2019, over periods of three alternating days during the hours of 6:00 p.m. to 8:00 p.m. Mosquito sampling was performed using automatic CDC and Shannon light traps. Canonical correspondence analysis (CCA), combined with Monte Carlo permutations, was used to evaluate the correlation between climatic variables and species distribution. In addition, non-metric multidimensional scaling (NMDS) was used to verify the similarity among the sampled communities from the different collections. After analyzing the total mosquito fauna at all sampling points, 46,564 specimens were identified, with *Mansonia dyari* showing the highest relative abundance in 2018 (35.9%). In contrast, *Mansonia titillans* had the highest relative abundance in 2019 (25.34%), followed by *Mansonia iguassuensis* (24.26%). The CCA showed that maximum temperature significantly influenced the distribution of mosquito populations in the study area (p = 0.0406). The NMDS showed that sampling carried out in the rainy and dry seasons formed two distinct groups. There was a significant correlation between species richness and cumulative precipitation 15 days before the sampling period (R^2^ = 58.39%; p = 0.0272). Thus, both temperature and precipitation affected mosquito population dynamics. The effect of rainfall on mosquito communities may be due to variations in habitat availability for immature forms.

## Introduction

Mosquito populations are dynamic, constantly changing over time according to factors that regulate their growth. Hence, studying the ecology of culicids in areas affected by large infrastructure projects is of fundamental importance. Specifically, understanding population dynamics can help elucidate community structure and interactions with the ecosystem, allowing us to answer questions, such as whether a population will persist in a particular habitat or not^1^. In the Amazon region, local ecosystem dynamics are affected by well-defined rainy and dry seasons^2^.

High temperatures associated with higher humidity and precipitation directly influence the life cycle of mosquitoes, favoring the development and survival of larvae, prolonging adult life, and thus increasing the overall population size^3–5^. Meanwhile, climate change accelerates the digestion of blood repasts performed by adult females, driving a higher intensity of hematophagous activity and, consequently, pathogen transmission.

Mosquitoes of the tribe Mansoniini tend to inhabit environments with a high degree of anthropogenic pressure. This proximity to the human population, in combination with remarkable resilience to new environments and the anthropophilic behavior of these mosquitoes, results in public health concerns^6^. Elevated levels of blood-feeding by populations of Mansoniini on animals and humans have caused disturbances to human life and livestock production in some regions^7^.

Therefore, studies of the bioecological and morphological configurations of the Mansoniini fauna are necessary to understand how possible environmental changes can affect the abundance and coexistence of mosquito species in the natural environment. Hence, this study aimed to evaluate the spatial distribution of mosquitoes in different seasons and the relationship between climatic factors and species abundance, focusing on populations of Mansoniini living in a region of the Brazilian Amazon affected by a hydroelectric plant.

## Materials and methods

### Ethics statement

Mosquito collections were authorized by the Chico Mendes Institute for Biodiversity Conservation—ICMBio, through the Biodiversity Authorization and Information System—SISBIO No.58855-3.

### Study area

The study area is located in the region surrounding the Jirau Hydroelectric Plant (HP), located 120 km from Porto Velho, in Rondônia state, Brazil, and is covered by vegetation of the Amazonian biome. The region’s landscapes vary according to local geographical particularities. Local vegetation types include Forested Wooded Campinarana, Shrubby Campinarana, Lowland Ombrophilous Forest with palm trees, Open Ombrophilous Forest, Várzea Forest, and Igapó Forest^8^. The state of Rondônia has an Aw-type tropical climate with dry winter (low rainfall in winter)^9^ and an average annual temperature of around 25.6 °C. The well-defined dry period of the winter season causes a moderate water deficit in the state, with rainfall rates below 50 mm/month^10^.

**Figure 1:**
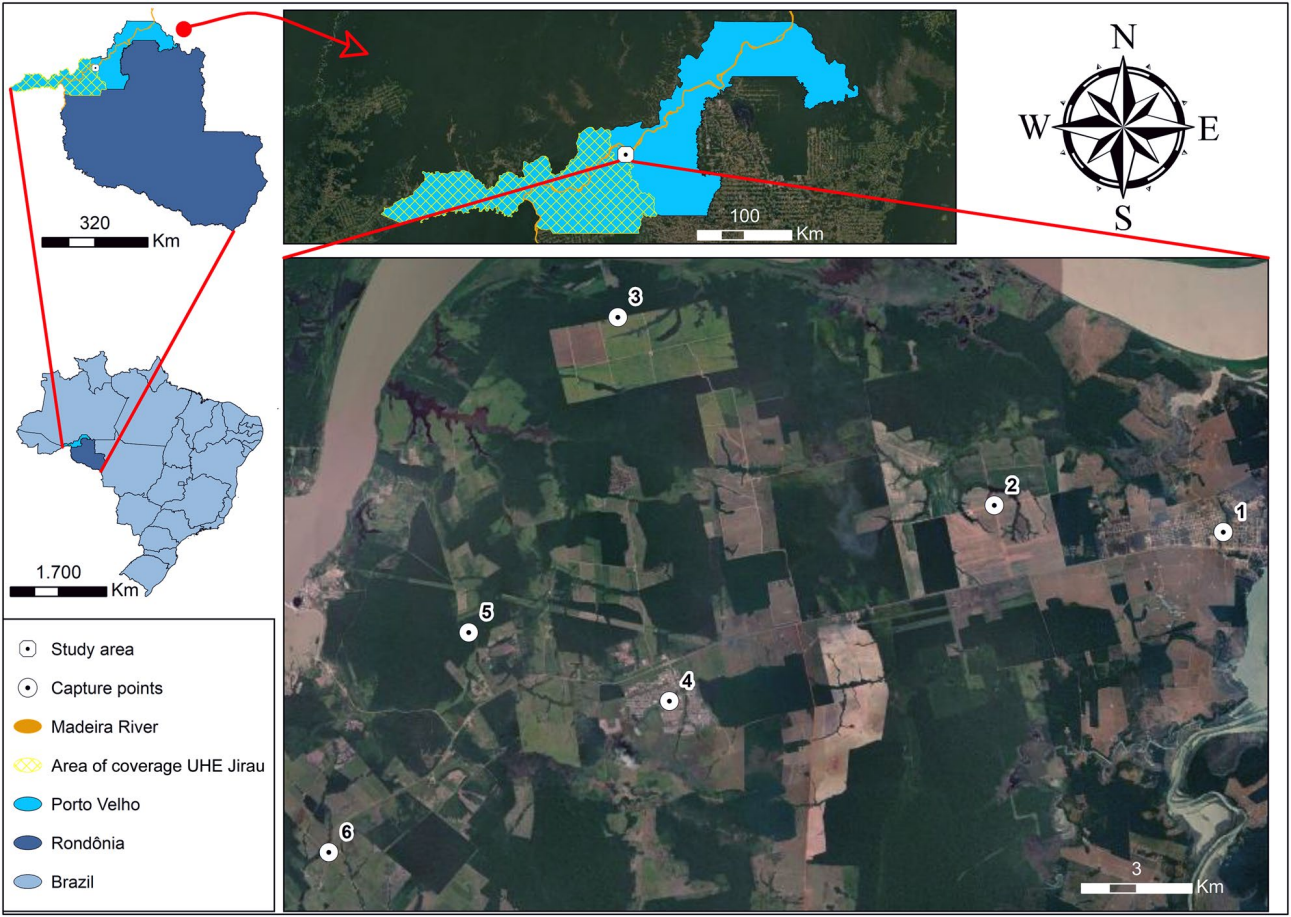
Sampling points, Jaci Paraná (1); Agrícola Zamo (2); Agrícola Zamo 2 (3); Nova Mutum Paraná (4); Right polygonal (5); Farm BR 364 KM 828 (6) located in the state of Rondônia, Brazil. *Source* Google Earth, satellite image/Pass date: June 2022.

Six sampling points were selected, situated between 3 and 25 km from the Jirau HP. These points were the Point 1, Jaci Paraná 9° 15′ 13.9′′ S 64° 24′ 44.7′′ W; Point 2, Agrícola Zamo 9° 14′ 50.3′′ S 64° 28′ 06.5′′ W; Point 3, Agrícola Zamo 2 9° 12′ 04.6′′ S 64° 33′ 37.8′′ W; Point 4, Nova Mutum Paraná 9° 17′ 42.5′′ S 64° 32′ 52.5′′ W; Point 5, Right polygonal 9° 16′ 42.1′′ S 64° 35′ 49.1′′ W; and Point 6, Farm BR 364 KM 828 9° 19′ 55.6′′ S 64° 37′ 52.3′′ W (Fig. 1). Sampling was carried out in May, July, October, and December 2018 and in April, July, September, and November 2019.

The captures were performed using CDC light traps for six consecutive days, from 06:00 p.m. to 09:00 a.m., for a total of 48 sampling days and a sampling effort of 720 h. We also used Shannon’s light traps from 6:00 p.m. to 8:00 p.m. for three alternate days, representing 24 additional samples with a sampling effort of 48 h. At the site used as the logistical support base for the study, all specimens captured in the Shannon and CDC traps were sacrificed with a chloroform solution, placed in conical polypropylene tubes with a layer of naphthalene and filter paper on the bottom, and preserved until identification. Each sample was identified by capture point, date, and sampling method. Species identification was carried out based on direct observation of the morphological characters under a stereomicroscope and consulting species descriptions/diagnoses in dichotomous keys by Refs.^7,11–14^. The abbreviation of the genera and subgenera followed the norms established by the Ref.^15^ group (2009). The captured specimens were listed in the Entomological Collection of the Oswaldo Cruz Institute under the title “Coleção Amazônica, UHE-JIRAU.”

### Statistical analysis

The correlations between the distribution of mosquito species and climatic variables were assessed through Canonical Correspondence Analysis (CCA), using CANOCO version 4.56. The statistical significance of the abiotic variables was tested with 5000 Monte Carlo permutations^16^.

Comparisons of the community structure found in different sampling campaigns were conducted through non-metric multidimensional scaling (NMDS) based on the Morisita index of similarity, using the Past 4.0 software. Using the Bioestat 5.3 computer program, a curve fitting was performed to assess the correlation between species richness and cumulative precipitation 15 days before each sampling period. This curve fitting indicated geometric regression as the best explanatory model for the data set.

Once the normal distribution of the data was ascertained using the Lilliefors test, Pearson’s correlation coefficients, with a 95% confidence interval, were calculated for correlations between total Culicidae species diversity and the diversity of *Mansonia* species, Mansoniini species, and *Coquillettidia* species. The Shannon–Wiener and Simpson indices were also used to describe the diversity of the species in the sampling area. The first index is more sensitive to variations in the number of rare species in the sample, and the second is more sensitive to variations in the most abundant species.

We used the Shannon index to evaluate the diversity of species found in the CDC and Shannon traps during the eight sampling periods of May, July, October, and December 2018, and April, July, September, and November 2019.

Measurements of relative air humidity and temperature (maximum, minimum, compensated average, and precipitation) were obtained from the National Institute of Meteorology^17^ and the National Agency of Water and Basic Sanitation^18^.

## Results

The mosquito fauna found in all sampling points of the study area was represented by the subfamilies Anophelinae and Culicinae, with a total of 46,564 adult mosquitoes captured from 32 species (Tables 1, 2).

**Table 1:** Absolute and relative abundance of Culicidae adults captured in areas surrounding the Jirau hydroelectric power plant in Rondônia state, Brazil, in May, July, October, and December 2018 and April, July, September, and November 2019.

Species/author	Campaigns and periods				AA*	RA**	Campaigns and periods				AA	RA**
	2018						2019					
	May	Jul	Oct	Dec			Apr	Jul	Sept	Nov		
	Dry	Dry	Transition	Rainy	N	%p	Transition	Dry	Dry	Rainy	N	%p
*Aedeomyia squamipennis* (Lynch Arribálzaga, 1878)	68	1	18	9	96	0.46	36	2	3	25	66	0.26
*Aedes fulvithorax* (Lutz, 1904)	1	0	0	0	1	0.00	0	0	0	0	0	0.00
*Aedes scapularis* (Rondani, 1848)	0	0	0	4	4	0.02	0	0	0	0	0	0.00
*Anopheles argyritarsis* Robineau-Desvoidy, 1827	0	0	0	0	0	0.00	0	0	1	0	1	0.00
*Anopheles evansae* (Brèthes, 1926)	0	0	0	0	0	0.00	1	0	0	0	1	0.00
*Anopheles triannulatus* (Neiva & Pinto, 1922)	109	0	1	3	113	0.54	0	0	2	2	4	0.02
*Coquillettidia albicosta* (Chagas, 1908)	1	0	0	1	2	0.01	0	0	0	1	1	0.00
*Coquillettidia albifera* (Prado, 1931)	0	0	0	1	1	0.00	0	0	0	0	0	0.00
*Coquillettidia chrysonotum* (Peryassú, 1922)	1	13	2	14	30	0.14	0	1	0	1	2	0.01
*Coquillettidia fasciolata* (Lynch Arribálzaga, 1891)	4	0	1	18	23	0.11	0	0	0	0	0	0.00
*Coquillettidia juxtamansonia* (Chagas, 1907)	1	7	4	5	17	0.08	0	0	1	2	3	0.01
*Coquillettidia lynchi* (Shannon, 1931)	4	1	0	1	6	0.03	0	0	1	1	2	0.01
*Coquillettidia nigricans* (Coquillett, 1904)	0	5	0	0	5	0.02	0	0	0	0	0	0.00
*Coquillettidia venezuelensis* (Theobald, 1912)	1	3	0	4	8	0.04	0	0	1	0	1	0.00
*Culex bastagarius* Dyar and Knab, 1906	46	3	5	41	95	0.46	3	0	0	96	99	0.38
*Culex (Melanonconion) *spp.	0	0	0	0	0	0.00	1	1	1	255	258	1.00
*Culex mollis* Dyar and Knab, 1906	55	2	6	96	159	0.77	30	11	0	19	60	0.23
*Mansonia amazonensis* (Theobald, 1901)	42	737	611	96	1486	7.16	495	671	1113	1405	3684	14.27
*Mansonia dyari *Belkin, Heinemann & Page, 1970	6193	1189	33	35	7450	35.91	24	6	96	0	126	0.49
*Mansonia flaveola* (Coquillett, 1906)	1	5	206	132	344	1.66	35	0	1	160	196	0.76
*Mansonia fonsecai* (Pinto, 1932)	1	59	281	73	414	2.00	44	174	14	73	305	1.18
*Mansonia humeralis* Dyar & Knab, 1916	284	757	107	263	1411	6.80	555	1893	161	2096	4705	18.22
*Mansonia iguassuensis *Barbosa, da Silva & Sallum, 2007	37	509	1063	493	2102	10.13	622	1933	2252	1456	6263	24.26
*Mansonia indubitans *Dyar & Shannon, 1925	99	448	91	232	870	4.19	159	75	50	90	374	1.45
*Mansonia pessoai* (Barreto & Coutinho, 1944)	1	4	0	1	6	0.03	2	0	0	35	37	0.14
*Mansonia pseudotitillans *(Theobald, 1901)	2	18	4	35	59	0.28	61	143	157	441	802	3.11
*Mansonia titillans* (Walker, 1848)	211	1781	1051	417	3460	16.68	1195	1147	1752	2448	6542	25.34
*Mansonia wilsoni* (Barreto & Coutinho, 1944)	1106	896	446	80	2528	12.19	246	475	694	868	2283	8.84
*Psorophora albipes* (Theobald, 1907)	0	0	0	9	9	0.04	0	0	0	0	0	0.00
*Psorophora cingulata* (Fabricius, 1805)	8	0	0	2	10	0.05	0	0	0	1	1	0.00
*Psorophora dimidiata* 1943 Cerqueira	3	0	0	0	3	0.01	0	0	0	0	0	0.00
*Psorophora ferox* (von Humboldt, 1819)	0	0	0	1	1	0.00	0	0	0	0	0	0.00
*Uranotaenia pulcherrima* Lynch Arribálzaga, 1891	33	0	0	0	33	0.16	0	0	0	2	2	0.01
Total	8312	6438	3930	2066	20,746	100	3509	6532	6300	9477	25,818	100

*Absolute abundance.

**Relative abundance.

**Table 2:** Abundance of adults of *Mansonia* spp., *Coquillettidia* spp., and other species captured using CDC and Shannon (SH) light traps near the Jirau hydroelectric plant in Rondônia state, Brazil, in 2018 and 2019.

	Campaigns	Season	No. of captured *Coquillettidia* spp.			No. of captured *Mansonia* spp.			Other species			Total No. of captured specimens (spp.)		
			CDC	SH	Total	CDC	SH	Total	CDC	SH	Total	CDC	SH	Total
2018	May	Dry	6	6	12	102	7875	7977	245	78	323	353	7959	8312
	July	Dry	2	27	29	422	5981	6403	5	1	6	429	6009	6438
	October	Transition	4	3	7	328	3565	3893	30	0	30	362	3568	3930
	December	Rainy	36	8	44	1463	394	1857	61	104	165	1560	506	2066
	Total		48	44	92	2315	17,815	20,130	341	183	524	2704	18,042	20,746
2019	April	Transition	0	0	0	542	2896	3438	69	2	71	611	2898	3509
	July	Dry	0	1	1	1375	5142	6517	14	0	14	1389	5143	6532
	September	Dry	0	3	3	263	6027	6290	5	2	7	268	6032	6300
	November	Rainy	4	1	5	3047	6025	9072	352	48	400	3403	6074	9477
	Total		4	5	9	5227	20,090	25,317	440	52	492	5671	20,147	25,818

In May, July, October, and December 2018, we captured 20,746 Culicidae specimens, of which 20,130 (97.03%) were from *Mansonia* species, and 92 (0.43%) were from *Coquillettidia*. Meanwhile, in April, July, September, and November 2019, we captured 25,818 Culicidae specimens, of which 25,317 (98.06%) were from *Mansonia* species and nine (0.03%) from *Coquillettidia*. In the months with the highest number of captures, we captured 9477 Culicidae specimens in November 2019, with 9072 (95.7%) from *Mansonia* spp., and 8312 in May 2018, of which 7977 (96%) belonged to *Mansonia* spp. (Table 1).

The highest number of Culicidae species was found in December 2018: 26 species, of which 18 belonged to Mansoniini (11 *Mansonia* spp.; 7 *Coquillettidia* spp.). In contrast, the month with the lowest number of species was July 2019, with 13, with 10 from Mansoniini (9 *Mansonia* spp.; 1 *Coquillettidia* spp.) (Table 1).

*Mansonia dyari* Belkin, Heinemann & Page, 1970 had the highest relative abundance (35.91%) in 2018 (Table 1). In 2019, meanwhile, *Mansonia titillans* (Walker, 1848) had the highest relative abundance, representing 25.34% of the sample, followed by *Mansonia iguassuensis* Barbosa, da Silva & Sallum, 2007, with 24.26% (Table 1). In total, 37,905 specimens of *Mansonia* spp. and 49 of *Coquillettidia* spp. were captured in Shannon light traps. In contrast, we captured 7542 *Mansonia* spp. and 52 *Coquillettidia* spp. in CDC traps (Table 2).

Of the 33 mosquito species identified, 28 were captured in the Shannon traps and 29 in the CDC traps. Four species occurred only in the Shannon trap and 5 in the CDC trap, with 24 species occurring in both traps. The numbers of Mansoniini captured were lowest in December 2018 (1901 specimens, or 4% of all specimens collected), April 2019 (3438; 8%), and October 2018 (3900; 9%) (Table 3).

**Table 3:** Abundance of species of the tribe Mansoniini in areas surrounding the Jirau hydroelectric plant in Rondônia state, Brazil, in May, July, October, and December 2018 and April, July, September, and November 2019.

Species/author	Sampling periods				AA*	RA**	Samplings periods				AA*	RA**
	2018						2019					
	May	Jul	Oct	Dec			Apr	Jul	Sept	Nov		
	Dry	Dry	Transition	Rainy	N	% p	Transition	Dry	Dry	Rainy	N	% p
*Coquillettidia albicosta* (Peryassú, 1908)	1	0	0	1	2	0.01	0	0	0	1	1	0.00
*Coquillettidia albifera* (Prado, 1931)	0	0	0	1	1	0.00	0	0	0	0	0	0.00
*Coquillettidia chrysonotum* (Peryassú, 1922)	1	13	2	14	30	0.15	0	1	0	1	2	0.01
*Coquillettidia fasciolata* (Lynch Arribálzaga, 1891)	4	0	1	18	23	0.11	0	0	0	0	0	0.00
*Coquillettidia juxtamansonia* (Chagas, 1907)	1	7	4	5	17	0.08	0	0	1	2	3	0.01
*Coquillettidia lynchi* (Shannon, 1931)	4	1	0	1	6	0.03	0	0	1	1	2	0.01
*Coquillettidia nigricans* (Coquillett, 1904)	0	5	0	0	5	0.02	0	0	0	0	0	0.00
*Coquillettidia venezuelensis* (Theobald, 1912)	1	3	0	4	8	0.04	0	0	1	0	1	0.00
*Mansonia amazonensis* (Theobald, 1901)	42	737	611	96	1486	7.35	495	671	1113	1405	3684	14.55
*Mansonia dyari *Belkin, Heinemann & Page, 1970	6193	1189	33	35	7450	36.84	24	6	96	0	126	0.50
*Mansonia flaveola* (Coquillett, 1906)	1	5	206	132	344	1.70	35	0	1	160	196	0.77
*Mansonia fonsecai* (Pinto, 1932)	1	59	281	73	414	2.05	44	174	14	73	305	1.20
*Mansonia humeralis* Dyar & Knab, 1916	284	757	107	263	1411	6.98	555	1893	161	2096	4705	18.58
*Mansonia iguassuensis *Barbosa, da Silva & Sallum, 2007	37	509	1063	493	2102	10.39	622	1933	2252	1456	6263	24.73
*Mansonia indubitans *Dyar & Shannon, 1925	99	448	91	232	870	4.30	159	75	50	90	374	1.48
*Mansonia pessoai* (Barreto & Coutinho, 1944)	1	4	0	1	6	0.03	2	0	0	35	37	0.15
*Mansonia pseudotitillans *(Theobald, 1901)	2	18	4	35	59	0.29	61	143	157	441	802	3.17
*Mansonia titillans* (Walker, 1848)	211	1781	1051	417	3460	17.11	1195	1147	1752	2448	6542	25,83
*Mansonia wilsoni* (Barreto & Coutinho, 1944)	1106	896	446	80	2528	12.50	246	475	694	868	2283	9.01
Total	7989	6432	3900	1901	20,222	100	3438	6518	6293	9077	25,326	100

*Absolute abundance.

**Relative abundance.

The canonical correspondence analysis revealed that only maximum temperature was a significant factor (p-value = 0.0406) with respect to the correlations between the abiotic variables (rainfall, temperature, and relative humidity) and the distributions of mosquito species in the sample area.

**Figure 2:**
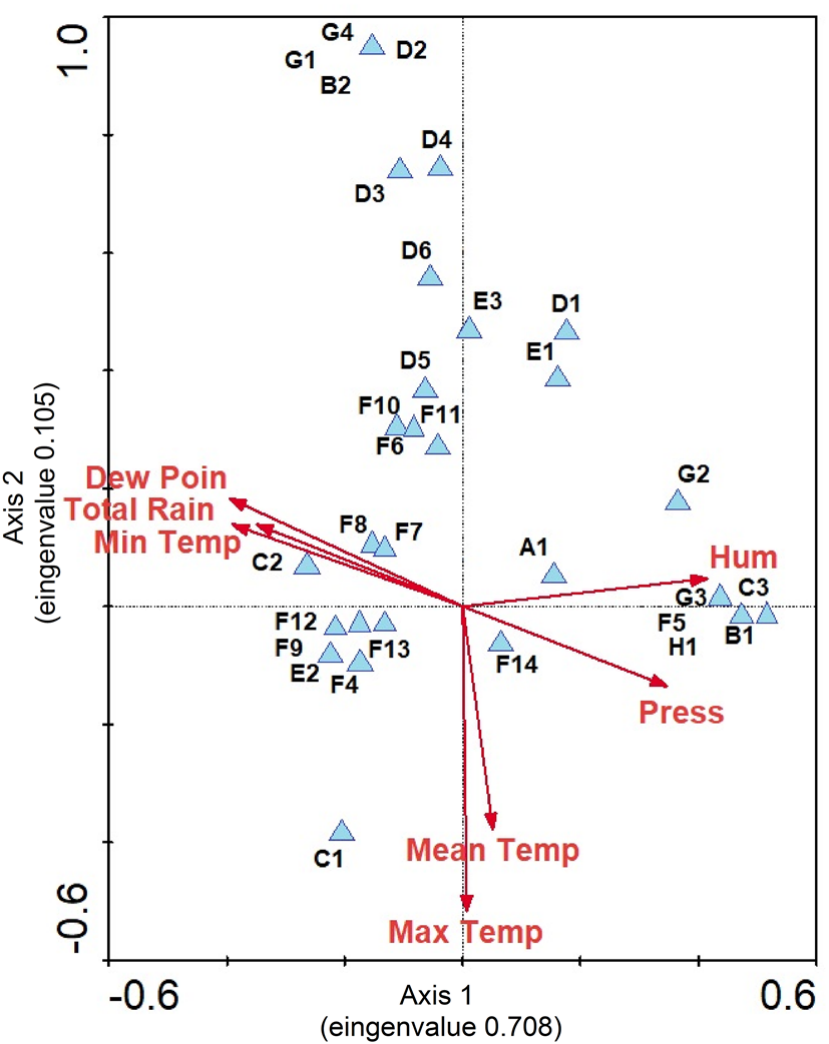
Ordering diagram generated by CCA (Axis 1 Eigenvalue = 0.708, Axis 2 Eigenvalue = 0.105) showing all mosquito species and climatic variables gathered during May, July, October, and December 2018 and April, July, September, and November 2019, near the Jirau hydroelectric plant in Rondônia state, Brazil. Only maximum temperature was found significant (p-value = 0.0406) after 5000 Monte Carlo permutations. A1: *Aedeomyia squamipennis;* B1: *Aedes fulvithorax;* B2: *Aedes scapularis;* C1: *Anopheles argyritarsis;* C2: *Anopheles evansae;* C3: *Anopheles triannulatus;* D1: *Coquillettidia albicosta; D2: Coquillettidia albifera;* D3: *Coquillettidia chrysonotum;* D4: *Coquillettidia fasciolata;* D5: *Coquillettidia juxtamansonia;* D6: *Coquillettidia venezuelensis;* E1: *Culex bastagarius;* E2: *Culex melanonconion;* E3: *Culex mollis*; F4: *Mansonia amazonensis;* F5: *Mansonia dyari;* F6: *Mansonia flaveola;* F7: *Mansonia fonsecai*; F8: *Mansonia humeralis;* F9: *Mansonia iguassuensis*; F10: *Mansonia indubitans*; F11: *Mansonia pessoai;* F12: *Mansonia pseudotitillans*; F13: *Mansonia titillans*; F14: *Mansonia wilsoni;* G1: *Psorophora albipes;* G2: *Psorophora cingulata*; G3: *Psorophora dimidiata;* G4: *Psorophora ferox;* H1: *Uranotaenia pulcherrima.*

Thus, Fig. 2 shows that all species in the lower two quadrants of the graph are positively influenced by the maximum temperature, while taxa in the upper two quadrants are negatively influenced.

**Figure 3:**
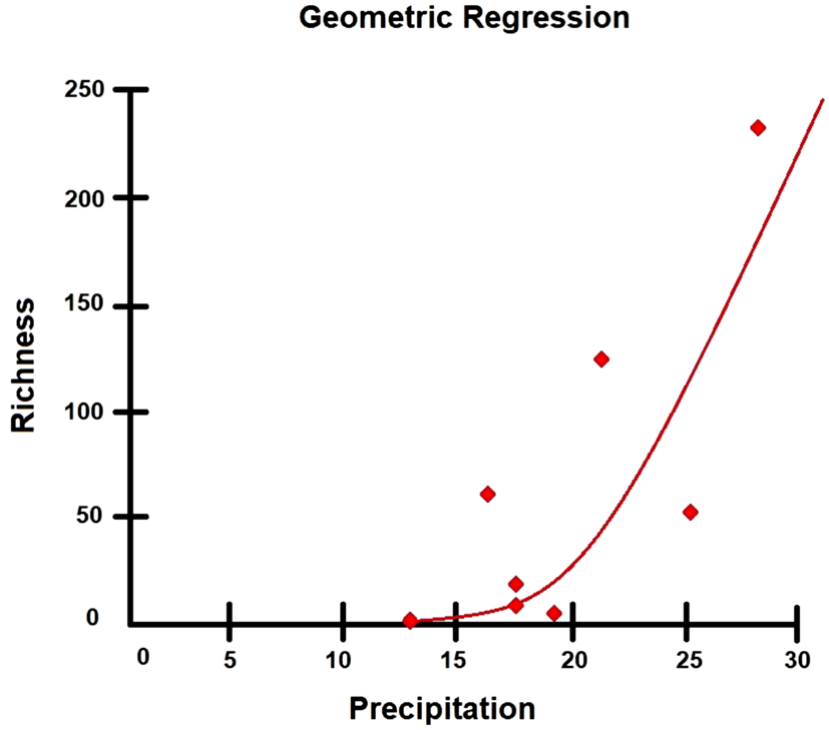
Regression curve of geometric regression (p-value = 0.0272, R^2^ = 58.39%) of species richness as a function of cumulative precipitation, indicating a positive correlation between these two variables.

The regression model with the best fit (R2 = 0.584; p = 0.0272) indicates a possible correlation between the cumulative rainfall prior to the sampling date and the number of species collected. Hence, a high rainfall intensity can directly influence mosquito abundance (Fig. 3).

The correlation between the diversity of *Mansonia* and that of all Culicidae was positive, strong, and highly significant (p = 0.0058, Pearson’s r = 0.8668), even more than the correlation between Mansoniini and the total Culicidae diversity, which was also significant (p = 0.0129, Pearson’s r = 0.8194). However, the correlation between the diversity of *Coquillettidia* and that of all culicid taxa was not significant.

**Figure 4:**
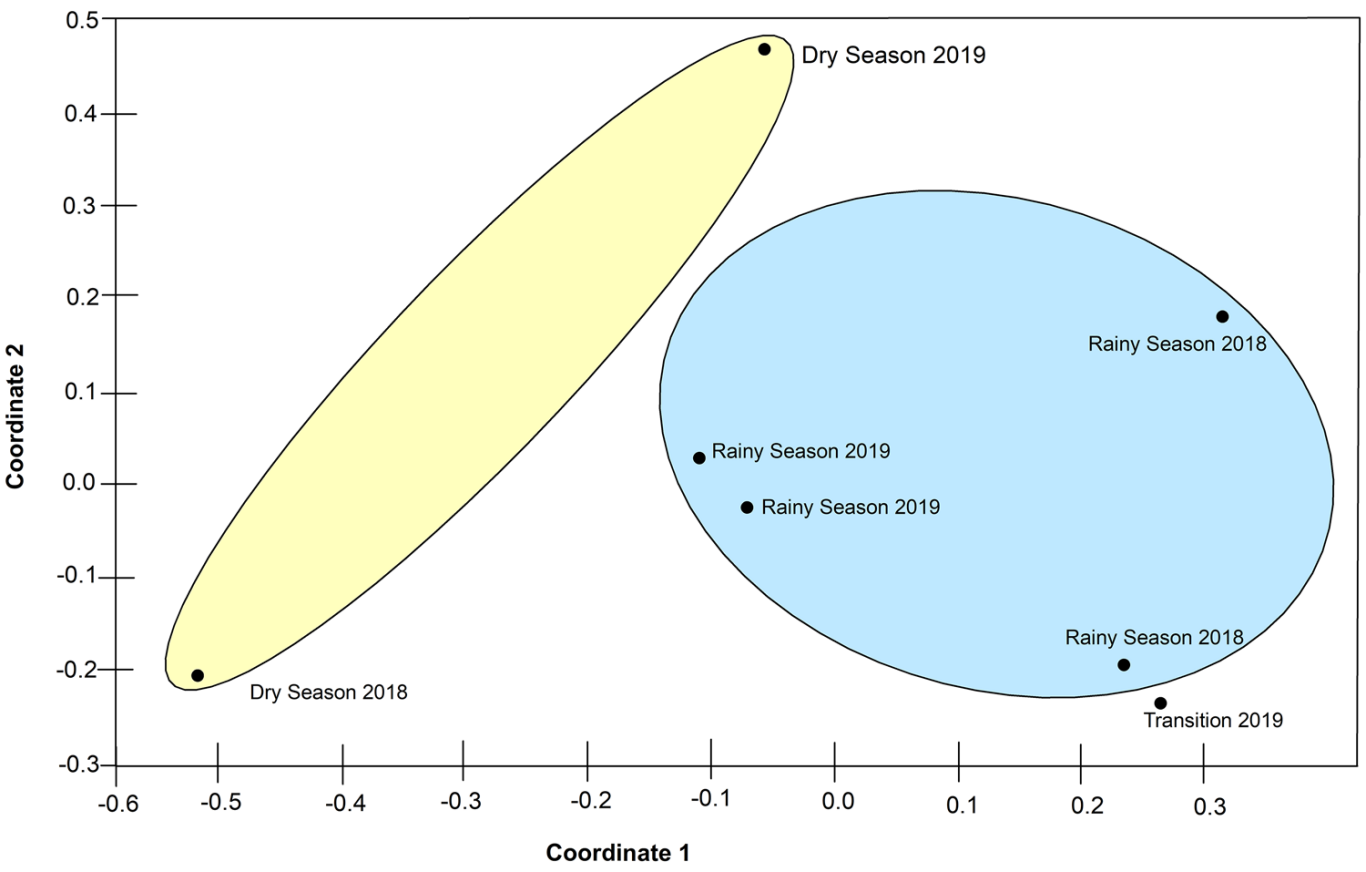
Non-metric multidimensional scaling (NMDS) using Morisita similarity index for ordination, depicting two distinct groups formed by collections from the wet and dry seasons (stress = 0.1071).

The NMDS revealed that the samples collected in the rainy season were highly similar, forming a set. In contrast, the collections of the dry season were less similar to each other, even forming a second group. Meanwhile, the collection carried out in the transition season showed a high degree of similarity with the sampling of the rainy season (Fig. 4). It should be noted that further statistical testing to quantify the effect of the climate season was limited as a result of the small sample size found in the dry season; although only two points were sampled during the dry season, their location in the reduced dimension space of the NMDS suggests that they differ from the samples of the rainy season.

Although the traps showed very similar diversity indices of captured species, the diversity was higher in CDC traps (2189) than Shannon traps (2003), even though the abundance of specimens was higher in Shannon traps (38,189) than the CDC traps (8375) (Table 4). However, when the Shannon index is calculated separately for Mansoniini, the Shannon traps show a higher level of diversity (1967) than the CDC light traps (1895) (Table 5).

**Table 4:** Abundance and Shannon diversity index of Culicidae adults captured in the CDC and Shannon traps installed near the Jirau hydroelectric plant in Rondônia state, Brazil, in 2018 and 2019.

Species/author	Traps					
	CDC		Shannon		Total	
	AA*	RA**	AA*	RA**	AA*	RA%
	N	%p	N	%p	N	%p
*Aedeomyia squamipennis* (Lynch Arribálzaga, 1878)	155	1.9	7	0.0	162	0.3
*Aedes fulvithorax* (Lutz, 1904)	1	0.0	0	0.0	1	0.0
*Aedes scapularis* (Rondani, 1848)	1	0.0	3	0.0	4	0.0
*Anopheles argyritarsis* Robineau-Desvoidy, 1827	1	0.0	0	0.0	1	0.0
*Anopheles evansae* (Brèthes, 1926)	0	0.0	1	0.0	1	0.0
*Anopheles triannulatus* (Neiva & Pinto, 1922)	52	0.6	65	0.2	117	0.3
*Coquillettidia albicosta* (Peryassú, 1908)	2	0.0	1	0.0	3	0.0
*Coquillettidia albifera* (Prado, 1931)	1	0.0	0	0.0	1	0.0
*Coquillettidia chrysonotum* (Peryassú, 1922)	18	0.2	14	0.0	32	0.1
*Coquillettidia fasciolata* (Lynch Arribálzaga, 1891)	17	0.2	6	0.0	23	0.0
*Coquillettidia juxtamansonia* (Chagas, 1907)	8	0.1	12	0.0	20	0.0
*Coquillettidia lynchi* (Shannon, 1931)	1	0.0	7	0.0	8	0.0
*Coquillettidia nigricans* (Coquillett, 1904)	0	0.0	5	0.0	5	0.0
*Coquillettidia venezuelensis* (Theobald, 1912)	5	0.1	4	0.0	9	0.0
*Culex bastagarius* Dyar & Knab, 1906	159	1.9	35	0.1	194	0.4
*Culex (Melanonconion)* spp.	226	2.7	32	0.1	258	0.6
*Culex mollis* Dyar & Knab, 1906	141	1.7	78	0.2	219	0.5
*Mansonia amazonensis* (Theobald, 1901)	1083	12.9	4087	10.7	5170	11,1
*Mansonia dyari *Belkin, Heinemann & Page, 1970	169	2.0	7407	19.4	7576	16.3
*Mansonia flaveola* (Coquillett, 1906)	309	3.7	231	0.6	540	1.2
*Mansonia fonsecai* (Pinto, 1932)	136	1.6	583	1.5	719	1.5
*Mansonia humeralis* Dyar & Knab, 1916	2570	30.7	3546	9.3	6116	13,1
*Mansonia iguassuensis *Barbosa, da Silva & Sallum, 2007	1229	14.7	7136	18.7	8365	18.0
*Mansonia indubitans *Dyar & Shannon, 1925	301	3.6	943	2.5	1244	2.7
*Mansonia pessoai* (Barreto & Coutinho, 1944)	8	0.1	35	0.1	43	0.1
*Mansonia pseudotitillans *(Theobald, 1901)	107	1.3	754	2.0	861	1.8
*Mansonia titillans* (Walker, 1848)	1348	16.1	8654	22.7	10,002	21.5
*Mansonia wilsoni* (Barreto & Coutinho, 1944)	282	3.4	4529	11.9	4811	10.3
*Psorophora albipes* (Theobald, 1907)	9	0.1	0	0.0	9	0.0
*Psorophora cingulata* (Fabricius, 1805)	0	0.0	11	0.0	11	0.0
*Psorophora dimidiata* Cerqueira de 1943	1	0.0	2	0.0	3	0.0
*Psorophora ferox* (von Humboldt, 1819)	0	0.0	1	0.0	1	0.0
*Uranotaenia pulcherrima* Lynch Arribálzaga, 1891	35	0.4	0	0.0	35	0.1
Total	8375	100.0	38,189	100.0	46,564	100.0
Shannon index	2189	–	2003	–	–	–

*Absolute abundance.

**Relative abundance.

**Table 5:** Abundance and Shannon diversity index of tribe Mansoniini specimens captured in the CDC and Shannon traps installed in areas near the Jirau hydroelectric plant in Rondônia state, Brazil, in 2018 and 2019.

Species/author	Traps					
	CDC		Shannon		Total	
	AA*	RA**	AA*	RA**	AA*	RA**
	N	%p	N	%p	N	%p
*Coquillettidia albicosta* (Peryassú, 1908)	2	0.0	1	0.0	3	0.0
*Coquillettidia albifera* (Prado, 1931)	1	0.0	0	0.0	1	0.0
*Coquillettidia chrysonotum* (Peryassú, 1922)	18	0.2	14	0.0	32	0.1
*Coquillettidia fasciolata* (Lynch Arribálzaga, 1891)	17	0.2	6	0.0	23	0.1
*Coquillettidia juxtamansonia* (Chagas, 1907)	8	0.1	12	0.0	20	0.0
*Coquillettidia lynchi* (Shannon, 1931)	1	0.0	7	0.0	8	0.0
*Coquillettidia nigricans* (Coquillett, 1904)	0	0.0	5	0.0	5	0.0
*Coquillettidia venezuelensis* (Theobald, 1912)	5	0.1	4	0.0	9	0.0
*Mansonia amazonensis* (Theobald, 1901)	1083	14.3	4087	10.8	5170	11,4
*Mansonia dyari *Belkin, Heinemann & Page, 1970	169	2.2	7407	19.5	7576	16.6
*Mansonia flaveola* (Coquillett, 1906)	309	4.1	231	0.6	540	1.2
*Mansonia fonsecai* (Pinto, 1932)	136	1.8	583	1.5	719	1.6
*Mansonia humeralis* Dyar & Knab, 1916	2570	33.8	3546	9.3	6116	13.4
*Mansonia iguassuensis *Barbosa, da Silva & Sallum, 2007	1229	16.2	7136	18.8	8365	18.4
*Mansonia indubitans *Dyar & Shannon, 1925	301	4.0	943	2.5	1244	2.7
*Mansonia pessoai* (Barreto & Coutinho, 1944)	8	0.1	35	0.1	43	0.1
*Mansonia pseudotitillans *(Theobald, 1901)	107	1.4	754	2.0	861	1.9
*Mansonia titillans* (Walker, 1848)	1348	17.8	8654	22.8	10,002	22.0
*Mansonia wilsoni* (Barreto & Coutinho, 1944)	282	3.7	4529	11.9	4811	10.6
Total	7594	100	37,954	100	45,548	100
Shannon index	1895	–	1967	–	–	–

*Absolute abundance.

**Relative abundance.

In particular, all species of Mansoniini were found in both traps, except *Coquillettidia albifera*, which was found only in the CDC light trap, and *Coquillettidia nigricans* (Coquillett, 1904)*,* found only in the Shannon trap. The highest Shannon diversity index for Mansoniini was observed in December 2018 (1990), during the rainy season in Rondônia. The month with the lowest diversity was May 2018 (0.816), considered part of the dry season. The months of July 2018 (dry), November 2019 (rainy), October 2018 (dry), and April 2019 (transition) also had very high diversity indices (Table 6).

**Table 6:** Shannon diversity index and Simpson index for adult Culicidae captured in areas near the Jirau hydroelectric plant in Rondônia state, Brazil, in 2018 and 2019.

Year	Campaigns	Period		Shannon index		Simpson index	
				Other culicidae	Mansoniini tribe	Other culicidae	Mansoniini tribe
2018	1	May	Dry	0.19070	0.81680	0.21863	0.62225
	2	July	Dry	0.00745	1.92783	0.38889	0.16844
	3	October	Transition	0.04516	1.83132	0.42889	0.19389
	4	December	Rainy	0.29976	1.99001	0.40731	0.16086
2019	5	April	Transition	0.09839	1.77767	0.43781	0.20823
	6	July	Dry	0.01458	1.68841	0.64286	0.22050
	7	September	Dry	0.00898	1.57986	0.30612	0.25059
	8	November	Rainy	0.13207	1.79041	0.00084	0.17222

The lowest Shannon indices were observed during the dry season months of May 2018 and September and July 2019. The month with the highest Simpson index (0.622) and, therefore, the lowest diversity was May 2018. In contrast, the month with the lowest Simpson index and, therefore, the highest diversity was December 2018 (0.160). These findings are consistent with those obtained by calculating the Shannon index (Table 6).

## Discussion

Changes in temperature and extreme environmental conditions can affect the dynamics of vector-borne pathogens. These include leishmaniasis, transmitted by phlebotomine sandflies, as well as mosquitoes that spread arboviruses like dengue, encephalitis, yellow fever, West Nile fever, and lymphatic filariasis^19–21^.

The CCA analysis showed that maximum temperature significantly influenced the abundance of mosquito populations in the study area. In addition, the NMDS showed two different groupings that consisted of samples collected during the rainy and dry seasons. Accordingly, Refs.^22,23^ report that changes in temperature and relative humidity determine the abundance of mosquitoes, which can disappear entirely during the dry season. Moreover, Refs.^22,24,25^ note that certain species of mosquitoes increase proportionally with the regional rainfall regime. This is consistent with Ref.^10^, who find alternating patterns in tropical and temperate climates in some Brazilian regions.

As shown by the geometric regression, there is a positive correlation between cumulative rainfall in the days before collection and the number of species found in the study period. Likewise, Ref.^26^ reported that under the conditions observed in the Serra do Mar State Park, climate variables directly influenced the abundance of *Cq. chrysonotum* and *Cq. venezuelensis*, favoring the occurrence of culicids during the more warm, wet, and rainy months.

The current climate scenario and future projections about climate, environmental, demographic, and meteorological factors directly influence the distribution and abundance of mosquito vectors and/or diseases^27–30^. Environmental temperature alters mosquito population dynamics, thereby affecting the development of immature stages as well as reproduction^31^. While temperature has an important effect on population dynamics, rainfall and drought also affect the density and dispersal of mosquitoes in temperate and tropical regions^32^.

To be sure, environmental changes other than climate can modify the behavior of vector insects and, subsequently, the mechanism of transmission of parasites^20^. Specifically, human impacts on the environment can result in drastically different disease transmission cycles in and around inhabited areas^33^.

A previous study^34^ reported that changes in land use influence the mosquito communities with potential implications for the emergence of arboviruses. Another study^35^ noted that environmental changes negatively affect natural ecosystems with accelerated biodiversity loss. This is due to the modification and loss of natural habitat and unsustainable land use, which leads to the spread of pathogens and disease vectors.

Hence, understanding the relationship between humans and the environment becomes increasingly critical, given the way in which climate changes can lead to alterations in the epidemiology of diseases such as dengue in areas considered free of the disease, as well as in endemic areas^36^.

We found that the abundance and diversity of Mansoniini were directly influenced by the effect of the rainy season and other climatic factors. The rainfall regime has been shown to affect the development of immature forms^12,37^; explaining the greater frequency of these specimens in the warmer and wetter months^38–40^. According to Ref.^41^, stable ecosystems such as forests contain great species diversity. On the other hand, diversity tends to be reduced in biotic communities suffering from stress.

Studies of insect populations in natural areas are important because they allow a direct analysis of how environmental factors influence phenomena such as the choice of breeding sites by females for oviposition, hematophagous behavior, and the distribution of species along a vegetation gradient^12,26,42,43^.

Throughout the experimental period of the present study, we observed that Shannon light traps are an effective method for catching mosquitoes from the Mansoniini tribe. Interestingly, Ref.^44^ reported a species richness pattern strongly influenced by *Coquillettidia fasciolata* (Lynch Arribálzaga, 1891) on mosquito samples from different capture points by using CDC and Shannon light traps as sampling methods. In contrast to the results of Ref.^44^, where the highest population density of mosquitoes was captured with CDC traps, we observed that these traps were not effective at capturing specimens of Mansoniini in spite of being used in large numbers in the present study. Moreover, Ref.^45^ conducted another study on faunal diversity in an Atlantic Forest remnant of the state of Rio de Janeiro and observed the highest abundance of *Cq. chrysonotum* (Peryassú, 1922) and *Cq. venezuelensis* by using Shannon light traps, while the numbers of captures of *Ma. titillans* were very similar using CDC and Shannon traps.

The results of this study indicate that the makeup of culicid fauna remains quite similar throughout the year, despite seasonal variations in abundance, though there was a lower variability of fauna in the dry season. Therefore, although the seasonality did not affect the temporal variation of the faunal composition in a generalized way, it was possible to detect a partial effect of the seasonality on fauna abundance.

Reference^46^ report that the incidence peaks of mosquitoes in the warmer and wetter months, as well as mosquito populations remaining between tolerance limits for most of the year, indicate the sensitivity of some species to the local climate.

The elevated abundance and diversity of species of Mansoniini in the study area were influenced by the favorable maintenance of breeding sites, including specific water accumulations with emerging vegetation that remain present throughout the year and the well-defined rainy season in the region. In addition, the representatives of Mansoniini, which prefer breeding sites containing macrophytes, made up nearly all of the species collected^7^.

Besides providing a greater awareness of mosquito populations’ ecological and biological aspects, research carried out in wild areas also provides information on the relationship between species diversity and the area in which they are found. Considering that wild insects may become potential vectors of diseases, research in wild areas also provides helpful information for understanding relevant epidemiological aspects. These studies facilitate the identification, monitoring, and control of mosquito populations following environmental changes caused by direct human action, which can lead to major epidemics^26^.

We observed considerable heterogeneity among Mansoniini fauna, and the months with the highest rainfall directly influence the structure of the communities and contribute to the increase in mosquito diversity and abundance, possibly due to variations in the availability of habitat for their immature forms.

## Data Availability

All data generated or analyzed during this study are included in this published article (and its Supplementary Information files).

## References

[1] Townsend CR, Begon M, Harper JL (2010). Fundamentos em Ecologia.

[2] de Souza EB (2009). Precipitação sazonal sobre a Amazônia oriental no período chuvoso: Observações e simulações regionais com o RegCM3. Rev. Bra. de Meteorol..

[3] Cator LJ (2020). The role of vector trait variation in vector-borne disease dynamics. Front. Ecol. Evol..

[4] Couper LI (2021). How will mosquitoes adapt to climate warming?. elife.

[5] Ezeakacha NF, Yee DA (2019). The role of temperature in affecting carry-over effects and larval competition in the globally invasive mosquito *Aedes albopictus*. Parasit. Vectors.

[6] Harbach, R. E. *Mosquito Taxonomic Inventory*. https://mosquito-taxonomic-inventory.myspecies.info/. Accessed 10 January 2022. (2022).

[7] Consoli RAGB, Oliveira RL (1994). Principais mosquitos de importância sanitária no Brasil.

[8] Fox JA, Brown LD, Fox JA (1998). The Struggle for Accountability: The World Bank, NGOs, and Grassroots Movements.

[9] Köeppen W (1948). Climatología: Con un estudio de los climas de la tierra.

[10] Mendonça F, Danni-Oliveira IM (2017). Climatologia: Noções básicas e climas do Brasil.

[11] Lane J (1953). Neotropical Culicidae.

[12] Forattini OP (2002). Culicidologia Médica: Identificação, Biologia, Epidemiologia.

[13] Barbosa, A. A. *Revisão do subgênero Mansonia Blanchard, 1901 (Diptera, Culicidae) e estudo filogenético de Mansoniini* Mestrado em Entomologia thesis, Universidade Federal do Paraná, (2007).

[14] Assumpção, I. C. *Chave de identificação pictórica para o subgênero Mansonia Blanchard, 1901 (Diptera, Culicidae) da região neotropical.* Bacharelado em Ciências Biológicas thesis, Universidade Federal do Paraná (2009).

[15] Reinert, J. F. *European Mosquito Bulletin* Vol. 27, 68–76 (2009).

[16] McCune B, Grace JB (2002). Analysis of Ecological Communities MjM Software.

[17] INMET. *Banco de Dados Meteorológicos Para ensino e Pesquisa*. https://portal.inmet.gov.br/. Acessed 7 November 2021. (2021).

[18] Terzian ACB (2015). Isolation and characterization of mayaro virus from a human in acre, Brazil. Am. J. Trop. Med. Hyg..

[19] Galati EAB, de Tamara NL, Natal D, Chiaravalloti-Neto F (2015). Mudanças climáticas e saúde urbana. Rev. USP..

[20] Franklinos LHV, Jones KE, Redding DW, Abubakar I (2019). The effect of global change on mosquito-borne disease. Lancet. Infect. Dis.

[21] Shocket MS, Ryan SJ, Mordecai EA (2018). Temperature explains broad patterns of Ross River virus transmission. Elife.

[22] Guimarães AÉ, Arlé M (1984). Mosquitos no Parque Nacional da Serra dos Órgãos, estado do Rio de Janeiro, Brasil: I-distribuição estacional. Mem. Inst. Oswaldo Cruz.

[23] de Melo Freire RC (2021). Ecological aspects of mosquitoes (Diptera: Culicidae) in a fragment of seasonal dry tropical forest (Caatinga) in Brazil. J. Arid Environ..

[24] Urcola JI, Fischer S (2019). Seasonal and environmental variables related to the abundance of immature mosquitoes in rain pools of a peri-urban park of Buenos Aires (Argentina). J. Med. Entomol..

[25] Bates M (1949). The Natural History of Mosquitoes.

[26] Guimarães AÉ, Gentile C, Lopes CM, Mello RPD (2000). Ecology of mosquitoes (Diptera: Culicidae) in areas of Serra do Mar State Park, State of São Paulo, Brazil. III-daily biting rhythms and lunar cycle influence. Mem. Inst. Oswaldo Cruz.

[27] Bartlow AW (2019). Forecasting zoonotic infectious disease response to climate change: Mosquito vectors and a changing environment. Vet. Sci..

[28] Ellwanger JH (2020). Beyond diversity loss and climate change: Impacts of Amazon deforestation on infectious diseases and public health. Anais da Acad. Bras. de Ciências..

[29] Brugueras S (2020). Environmental drivers, climate change and emergent diseases transmitted by mosquitoes and their vectors in southern Europe: A systematic review. Environ. Res..

[30] Fouque F, Reeder JC (2019). Impact of past and on-going changes on climate and weather on vector-borne diseases transmission: A look at the evidence. Infect. Dis. Poverty.

[31] Couret J, Benedict MQ (2014). A meta-analysis of the factors influencing development rate variation in *Aedes aegypti* (Diptera: Culicidae). BMC Ecol..

[32] Elbers ARW, Koenraadt CJ, Meiswinkel R (2015). Mosquitoes and Culicoides biting midges: Vector range and the influence of climate change. Rev. Sci. Tech..

[33] Colón-González FJ (2021). Projecting the risk of mosquito-borne diseases in a warmer and more populated world: A multi-model, multi-scenario intercomparison modelling study. Lancet Planet. Health.

[34] da Silva Pessoa Vieira CJ (2022). Land-use effects on mosquito biodiversity and potential arbovirus emergence in the Southern Amazon, Brazil. Transbound. Emerg. Dis..

[35] Alho CJR (2012). Importância da biodiversidade para a saúde humana: Uma perspectiva ecológica. Estudos Avançados.

[36] Barcellos C (2009). Mudanças climáticas e ambientais e as doenças infecciosas: Cenários e incertezas para o Brasil. Epidemiol. e Serv. de Saúde.

[37] Casanova C, Do Prado AP (2002). Key-factor analysis of immature stages of *Aedes scapularis* (Diptera: Culicidae) populations in southeastern Brazil. Bull. Entomol. Res..

[38] Teodoro U (1994). Mosquitos de ambientes peri e extradomiciliares na região sul do Brasil. Rev. Saude Publica.

[39] Freitas Silva SO, de Mello CF, Machado SL, Leite PJ, Alencar J (2022). Interaction of *Haemagogus leucocelaenus* (Diptera: Culicidae) and other mosquito vectors in a forested area, Rio de Janeiro, Brazil. Trop. Med. Infect. Dis..

[40] La Corte R, Maia PCR, Dolabella SS, Cruz DER, Marteis LS (2019). Mosquitoes of the Caatinga. III. Larval habitats, frequency, and dynamics of immature and adult stages in a dry Brazilian forest. J. Med. Entomol..

[41] Richardson BA (1999). The bromeliad microcosm and the assessment of faunal diversity in a Neotropical forest. Biotropica.

[42] Guimarães AÉ, Gentile C, Lopes CM, Sant'Anna A, Jovita AM (2000). Ecologia de mosquitos (Diptera: Culicidae) em áreas do Parque Nacional da Serra da Bocaina, Brasil. I-Distribuição por habitat. Rev. Saude Publica.

[43] Guimarães AÉ, Mello RPD, Lopes CM, Gentile C (2000). Ecology of mosquitoes (Diptera: Culicidae) in areas of Serra do Mar State Park, State of São Paulo, Brazil. I-monthly frequency and climatic factors. Mem. Inst. Oswaldo Cruz.

[44] Alencar J (2012). Evaluation of mosquito (Diptera: Culicidae) species richness using two sampling methods in the hydroelectric reservoir of Simplicio, Minas Gerais, Brazil. Zool. Sci..

[45] Alencar J (2020). Ecosystem diversity of mosquitoes (Diptera: Culicidae) in a remnant of Atlantic Forest, Rio de Janeiro state, Brazil. Austral Entomol..

[46] Guimarães AÉ, Gentile C, Catarina ML, Sant’Anna A (2001). Ecologia de mosquitos em áreas do Parque Nacional da Serra da Bocaina: II—Freqüência mensal e fatores climáticos. Rev. de Saúde Pública.

